# Antioxidative Effects and Inhibition of Human Low Density Lipoprotein Oxidation *In Vitro* of Polyphenolic Compounds in *Flammulina velutipes* (Golden Needle Mushroom)

**DOI:** 10.1155/2015/403023

**Published:** 2015-06-09

**Authors:** Mohammad Azizur Rahman, Noorlidah Abdullah, Norhaniza Aminudin

**Affiliations:** ^1^Mushroom Research Centre, Institute of Biological Sciences, Faculty of Science, University of Malaya, 50603 Kuala Lumpur, Malaysia; ^2^Department of Biochemistry and Molecular Biology, Jahangirnagar University, Savar, Dhaka 1340, Bangladesh

## Abstract

Dietary polyphenolic compounds mediate polynomial actions in guarding against multiple diseases. Atherosclerosis is an oxidative stress driven pathophysiological complication where free radical induced oxidative modification of low density lipoprotein (LDL) plays the ground breaking role. Mushrooms have been highly regarded for possessing an antioxidant arsenal. Polyphenolic compounds present in dietary mushrooms seem pertinent in withstanding LDL oxidation en route to controlling atherosclerosis. In this study, the antioxidative effect of five solvent fractions consisting of methanol : dichloromethane (M : DCM), hexane (HEX), dichloromethane (DCM), ethyl acetate (EA), and aqueous residue (AQ) of *Flammulina velutipes* was evaluated. M : DCM fraction showed the most potent 2,2-diphenyl-1-picrylhydrazyl radical scavenging effect with IC_50_ of 0.86 mg/mL and total phenolic content of 56.36 gallic acid equivalent/g fraction. In LDL oxidation inhibitory tests, M : DCM fraction at 1 *µ*g/mL concentration mostly lengthened the lag time (125 mins) of conjugated diene formation and inhibited the formation of thiobarbituric acid reactive substances (48.71%, at 1 mg/mL concentration). LC-MS/MS analyses of M : DCM fraction identified the presence of polyphenolic substances protocatechuic acid, *p*-coumaric, and ellagic acid. These chain-breaking polyphenolics might impart the antioxidative effects of *F. velutipes*. Thus, mushroom-based dietary polyphenolic compounds might be implicated in slowing down the progression of atherosclerosis.

## 1. Introduction

The implication of dietary polyphenols and their effects upon cell biochemistry and pathophysiology emanates from their prophylactic role against multiple diseases. Polyphenols entail a diverse array of plant and fungal secondary metabolites having pluripotent actions at the cellular level [1]. Their mode of action is as diverse as their structures are and both ameliorating and null effects have been noticed while considering antioxidative role of dietary polyphenols [2, 3]. Substantial evidences support their involvement in cellular signaling processes that ultimately result in reduced production of inflammatory mediators and increased generation of vasodilators [4, 5]. Although no direct antioxidative function of dietary polyphenols has been noticed in human cardiovascular health studies, polyphenols have been implicated in inducing antioxidant defense by stimulating the Nrf2/Keap1 signaling pathway [6, 7].

Actually, free radicals are being constantly produced in our body as byproducts or defensive weapons against pathogens and also removed accordingly with a view to maintaining normal cellular biochemistry and physiology [8, 9]. However, the imbroglio of their excessive production and accumulation afflicts the cellular and physiological normalcy resulting in pathophysiological consequences [10]. Atherosclerosis being such a free radical-induced and oxidative stress-governed predicament imparts the major morbidity and mortality associated with cardiovascular diseases (CVD) [11]. Etiologically, free radicals oxidatively attack the low density lipoproteins (LDL) and the oxidized LDL molecules (ox-LDL) become modified both structurally and functionally [12]. This occurs in the arterial wall and the ox-LDL manifests multiple proatherogenic potential including its stimulatory role towards excessive production of proinflammatory cytokines, formation of foam cells, impairment of the endothelial vasculature, and endothelial dysfunction [13]. The ox-LDL itself is cytotoxic towards endothelial cells as it generates many free radicals only to worsen the atherogenic situation [14]. Thus, controlling of atherosclerosis warrants proactive action of therapeutic strategies capable of slowing down the generation of ox-LDL.

Prodigious strive for mitigating oxidative modification of LDL has flared towards boosting antioxidative defense arsenal up. Natural food and nutraceutical-based approaches come forward in this regard [15]. Edible macrofungi have been highly recognized for possessing numerous bioactive components of both nutritional and medicinal importance [16, 17]. Among different varieties,* Flammulina velutipes* (Curtis) Singer (also known as the golden needle mushroom, lily mushroom, enokitake, enokidake, and/or enoki) is a white, thin, and long mushroom species. It is popular as a delicate cuisine. Its immune-stimulating effects have been well recognized and the associated fungal immune-modulatory protein (FIP), FVE, has been reported [18, 19]. Its immune-modulating polysaccharides have also been identified and structure-activity relationship has been elucidated [20, 21]. Compared to other mushroom species, its antioxidative status and corresponding implication upon atherosclerosis have been less documented. Thus,* Flammulina velutipes* had been chosen under the present study to evaluate its effect upon* in vitro* atherosclerosis attenuation through mitigation of* in vitro* oxidative stress followed by the identification of responsible bioactive components, especially the polyphenolics. To our knowledge, this is the first report indicating the presence of polyphenolic compounds in* F. velutipes* solvent partitioned fractions and relating those seminal biocomponents with antiatherosclerotic venture.

## 2. Materials and Methods

### 2.1. Solvent Partitioning and Preparation of Liquid-Liquid Fractions

Mushroom bioactive components warrant appropriate separation, recovery, and purification processes for obtaining maximum output from their usage. Solvent partitioning, also called liquid-liquid partitioning, is a steady-state procedure for fractioning biocomponents [22]. It enables the separation of biocompounds based on their relative solubility in two different immiscible solvents [23]. In the present study, the modified method of Mayakrishnan et al. (2013) was applied for solvent partitioning and fractionation of* F. velutipes *[24]. Briefly, each two-hundred-gram powder was fractioned with 4 L of methanol : dichloromethane (M : DCM) (2 : 1) using conical flasks at room temperature with occasional stirring and shaking, followed by filtration through Whatman number 1 filter paper. The total organic solution was evaporated using a rotary evaporator (Büchi Rotavapor R-114, Switzerland) that yielded the M : DCM fraction. An aliquot of M : DCM fraction was dissolved in 90% aqueous methanol and partitioned with hexane (3 × 100 mLs). Separating funnel-based assay of the upper hexane layer was followed by the rotaevaporation of hexane and the hexane fraction collected. The aqueous methanolic layer left at the bottom was rotaevaporated and the semisolid fraction redissolved in distilled water (100 mL). Then successive partitioning with dichloromethane (DCM, 3 × 100 mLs) and rotaevaporation resulted in the DCM fraction. The bottom-layered aqueous fraction was repartitioned with ethyl acetate (EA, 3 × 100 mLs). Ethyl acetate was rotaevaporated and the lowered aqueous part freeze-dried to gain the respective fraction.

### 2.2. Sources of the Chemicals

Analytical grade chemicals were used in the present study. All of them had been purchased from Sigma-Aldrich (USA).

### 2.3. Investigation into the Antioxidant Activities of the Fractions

The following standard assays were performed to delve out the antioxidant potentiality of the various fractions of* F. velutipes *and compared with the natural antioxidant, quercetin.

#### 2.3.1. Scavenging Effect on 1,1-Diphenyl-2-picrylhydrazyl (DPPH) Radical

The method previously described by Abdullah et al. (2012) was followed [25]. In short, 0.1 mL of each solvent fraction (1.0 mg/mL conc.) was mixed with 3.9 mL of 0.06 mM DPPH dissolved in methanol. The solution was shaken vigorously under darkness and absorbance was taken at 515 nm. Methanol was used as the blank. The percentage of DPPH free radical scavenging was calculated using the following equation:

(1) $$\begin{array}{c} \text{radical}\;\text{scavenging}\;\text{activity}\left(\%\right)=\frac{\left(A_{0}-A_{s}\right)}{A_{0}}\times 100, \end{array}$$

where *A*_0_ is the absorbance of the 0.06 mM methanolic DPPH only and *A*_*s*_ is the absorbance of the reaction mixture. IC_50_ value (concentration of the fraction to produce half maximal inhibition/scavenging) of the most potent solvent fraction was calculated from the graph of the radical scavenging activity against fraction concentration.

#### 2.3.2. Folin-Ciocalteu Assay

The modified methodology of Slinkard and Singleton (1977) was used to determine the reducing potential of the fractions [26]. In brief, 250 *μ*L of each of the solvent fractions was added to an equal volume of 10% Folin-Ciocalteu reagent and kept at darkness for 3 minutes while shaking. Then, 500 *μ*L of 10% sodium carbonate was added to the reagent mixture and kept at dark again. After 1 h, absorbance was taken at 750 nm. The calibration curve of gallic acid (2–10 *μ*g/mL) was used to express the performance of the Folin-Ciocalteu assay as gallic acid equivalents (GAE) per gram of fraction.

#### 2.3.3. Inhibition of Lipid Peroxidation of Buffered Egg Yolk

A slightly modified version of the method described by Daker et al. (2008) was used to determine the inhibitory effect of each of the fractions on lipid peroxidation of the buffered egg yolk [27]. In a nutshell, 0.5 g of fowl egg yolk was emulsified with 0.1 M phosphate buffer (pH 7.4) so that the final volume becomes 25 g/L. Ferrous sulphate (1 M, 100 *μ*L) was added to the mixture to induce lipid peroxidation. Thereafter, fractions of various concentrations were introduced to the peroxidation-prone milieu at the volume of 100 *μ*L and shaken vigorously. After 1 h incubation at room temperature, 0.5 mL of 15% trichloroacetic acid (TCA) and 1 mL of thiobarbituric acid (TBA), both freshly prepared, were added. After incubation on the boiling water bath for 10 minutes, the tubes containing the reaction mixtures were cooled at room temperature and centrifuged at 3,500 g for 10 minutes to precipitate the proteins. Supernatant (100 *μ*L) was taken to measure the formation of thiobarbituric acid reactant substances (TBARS) through studying absorbance at 532 nm. Buffered egg yolk with Fe^+2^ only was used as the control. The percentage inhibition of lipid peroxidation was calculated using the following equation:

(2) $$\begin{array}{c} \text{inhibition}\left(\%\right)=\frac{\left(A_{0}-A_{s}\right)}{A_{0}}\times 100, \end{array}$$

where *A*_0_ is the absorbance of the control and *A*_*s*_ is the absorbance of the reaction mixture containing the fraction. IC_50_ value (concentration of the fraction to produce half maximal inhibition) of the most potent solvent fraction was calculated from the graph of the inhibition of lipid peroxidation against fraction concentration.

#### 2.3.4. FeSO_4_-Induced LDL Oxidation and* F. velutipes* Fractions-Mediated Inhibition

Two features of LDL oxidation were measured: lag time extension during conjugated diene (CD) formation and inhibition of thiobarbituric acid reactive substances (TBARS) production.

*(a) Measurement of the Lag Time Extension during CD Formation*. The method described by Rahman et al. (2014) was applied to measure the fractions' inhibitory effect on the human LDL oxidation through extension of lag period of CD formation [9]. Human LDL concentration was adjusted to 150 *μ*g/mL and the reaction volume totaled to 200 *μ*L with 0.1 M phosphate buffer, pH 7.4. Transition metal ion induced oxidative stress to LDL was exerted by freshly prepared FeSO_4_ solution (50 *μ*g/mL) at room temperature. LDL oxidation kinetics of Fe^+2^ and antioxidant effect of the fractions were studied at 234 nm at 20-minute intervals for a period of 3 h. FeSO_4_ in ultrapure water only, at pH 7.4, was used as the blank.* F. velutipes* fractions-mediated protection period of the LDL oxidation was dubbed as the “lengthened lag time of CD formation” and was measured until the amount of the CD began to increase.

*(b) Inhibitory Effect upon the Formation of TBARS*. For determining the inhibitory effects of* F. velutipes* upon TBARS formation, the method developed by Rahman et al. (2014) was employed [9]. Ferrous sulphate (10 mM, 191 *μ*L) was applied upon human LDL (9 *μ*L) to generate oxidative modification of LDL.* F. velutipes* fraction at 1 *μ*g/mL (100 *μ*L) was added with the oxidized LDL. Freshly prepared 500 *μ*L of 15% trichloroacetic acid (TCA) and 1 mL of 1% thiobarbituric acid (TBA) were added and incubated at 100°C for 10 minutes followed by cooling at room temperature. Finally, an aliquot (300 *μ*L) was taken to the ELISA reader and the absorbance read at 532 nm. For blank, FeSO_4_ in water, pH 7.4, was used. The percentage inhibition of TBARS formation was calculated using the following equation:

(3) $$\begin{array}{c} \text{inhibition}\left(\%\right)=\frac{\left(A_{0}-A_{s}\right)}{A_{0}}\times 100, \end{array}$$

where *A*_0_ is the absorbance of the control and *A*_*s*_ is the absorbance of the reaction mixture containing the fraction. IC_50_ value (concentration of the fraction to produce half maximal inhibition) of the most potent solvent fraction was calculated from the graph of the inhibition of TBARS against fraction concentration.

#### 2.3.5. Identification of Polyphenols and Other Bioactive Components by LC-MS/MS

Liquid chromatography-mass spectroscopy (LC-MS/MS) full scan analysis was performed to determine the presence of polyphenolic compounds in the M : DCM fraction of* F. velutipes.* The system setup involved ionization mode: negative; column: Zorbax C18, 150 mm × 4.6 mm × 5 *μ*M; buffer: (a) water with 0.1% formic acid and 5 mM ammonium formate and (b) acetonitrile with 0.1% formic acid and 5 mM ammonium formate; run time: 15-minute rapid screening. The apparatus utilized was AB Sciex 3200QTrap LCMS/MS with Perkin Elmer FX 15 uHPLC System (Perkin Elmer, USA). MS setting included voltage IS: −4500 V, source temperature: 500°C, desolvation gas: 40 psi, source gas: 40 psi, scan range: 100–1200* m/z* for full scan and 50–1200* m/z* for MS/MS scan, declustering potential: 40 V, entrance potential: 10 V, and collision energy: spread of 35 eV ± 15 eV. Sample fractions were diluted in appropriate solvent such as methanol, dichloromethane and substitution with acetonitrile in preparation for LCMS/MS analysis, filtered with nylon 0.22 *μ*M, and injected at volume 20 *μ*L.

#### 2.3.6. Statistical Analyses

We conducted all the experiments in triplicate and presented the data as mean ± SD. Using statistical package SPSS version 16, we performed one-way analysis of variance (ANOVA). The differences among means were further analyzed by least significance difference (LSD) test at 95% level (*P* ≤ 0.05).

## 3. Results and Discussion

### 3.1. DPPH Free Radical Scavenging Activity

**Figure 1:**
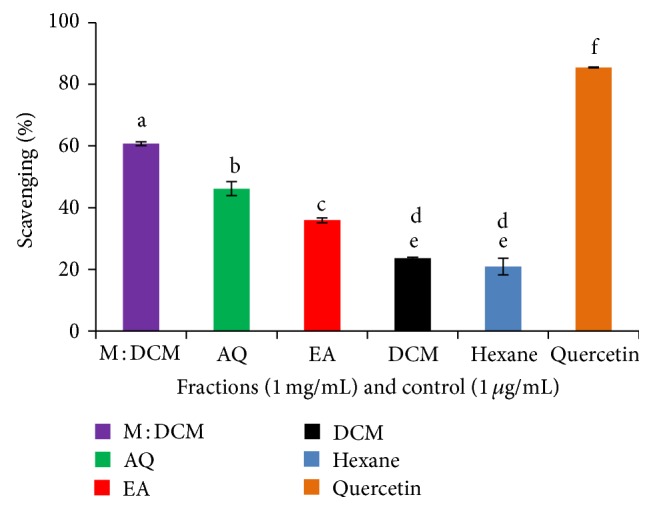
DPPH free radical scavenging effect of the* F. velutipes* fractions compared to the positive control, quercetin. Data presented as mean ± SD of triplicate determinations. Mean values with different lowercase superscripts (a–f) represent statistically significant difference at 95% level (*P* ≤ 0.05) with post hoc least significance difference (LSD) test.

The antioxidant property of natural compounds was most commonly evaluated on the basis of DPPH free radical scavenging property. Although the test is not any single antioxidant-specific but rather measures the cumulative antioxidant potential of any sample, the procedure is simple, rapid, and cost effective. Specifically, the scavenging antioxidants reduce the stable, purple radical DPPH^∙^ through bond pairing between its odd electron and scavenging antioxidant's hydrogen atom and convert it into yellow colored, nonradical form (DPPH-H). As shown in Figure 1, at 1 mg/mL concentration, the methyl : DCM fraction of* F. velutipes* performed the best DPPH free radical scavenging effect (60.75%, IC_50_ 0.86 mg/mL).

### 3.2. Folin-Ciocalteu Assay

**Figure 2:**
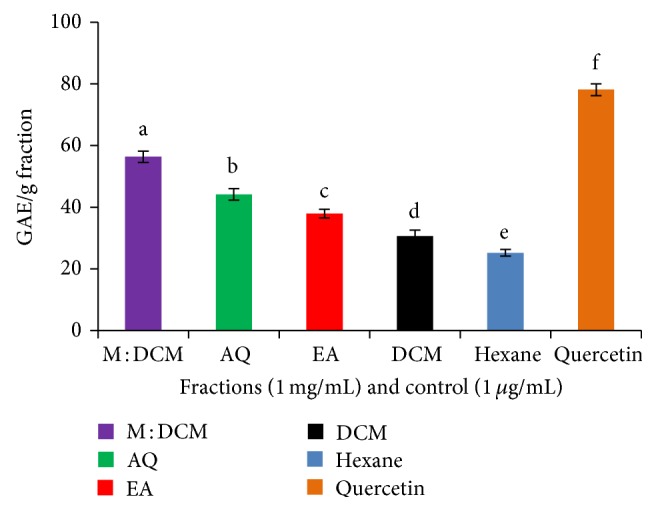
Folin-Ciocalteu assay of the* F. velutipes* fractions compared to positive control, quercetin. Data presented as mean ± SD of triplicate determinations. Mean values with different lowercase superscripts (a–f) represent statistically significant difference at 95% level (*P* ≤ 0.05) with post hoc least significance difference (LSD) test.

The antioxidative prowess of mushrooms depends largely on their biochemical composition involving phenolics, flavonoids, carotenoids, ascorbic acid, amino acids, and various enzymes that impart reducing effect on the oxidant-laden molecules or ions. Through Folin-Ciocalteu assay, the reducing capacities of each of the fractions had been measured and the results had been expressed as gallic acid equivalents (GAE) per gram of the dried fraction. As is evident from Figure 2, the M : DCM fraction contained the highest reducing capacity (56.36 GAE/g fraction), followed by that of the aqueous one (44.16 GAE/g fraction).

### 3.3. Lipid Peroxidation Inhibition Test

**Figure 3:**
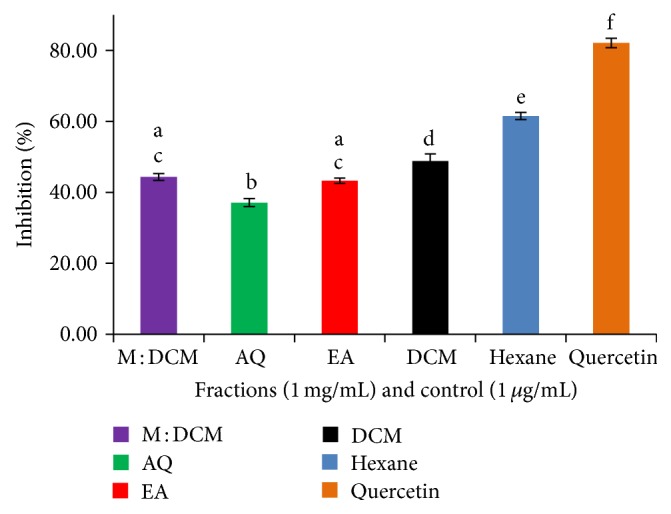
Lipid peroxidation inhibitory effect of* F. velutipes* fractions compared to positive control, quercetin. Data presented as mean ± SD of triplicate determinations. Mean values with different lowercase superscripts (a–f) represent statistically significant difference at 95% level (*P* ≤ 0.05) with post hoc least significance difference (LSD) test.

The peroxidative modification of lipid structures and the resulting products are among the key factors initiating the pathogenesis of atherosclerosis. The unsaturated portions of lipids, especially the double bonds of fatty acids present in lipid molecules, are most vulnerable to oxidative attack by free radicals and ions that lead to altered lipid structures resulting in the breakdown products like malondialdehyde (MDA). Thus, estimation of whether mushroom fractions could inhibit, albeit reduce, the formation of malondialdehyde, equivalent to the reduced lipid peroxidation, would be of high importance in assessing the antioxidative potential of the mushroom fractions in concern [28]. Based on this paradigm, we induced egg yolk lipid peroxidation by Fe^+2^ at low pH and elevated temperature and tested the action of each fraction. We found that the hexane fraction had the most potent inhibitory effect on lipid peroxidation (61.52%), followed by DCM (48.79%). M : DCM fraction stood third in position (44.33%) (Figure 3). The reason may be that the polar, hydrophilic polyphenolics were not in appropriate amount to baffle the nonpolar, hydrophobic milieu of the lipid peroxidation. Similarly, the high inhibitory effect of the nonpolar hexane fraction might be attributed to its lipophilic and/or biocomponents contents.

### 3.4. Inhibition of LDL Oxidation by* F. velutipes* Fractions

#### 3.4.1. Effect of* F. velutipes* Fractions upon Lag Time of CD Formation

**Figure 4:**
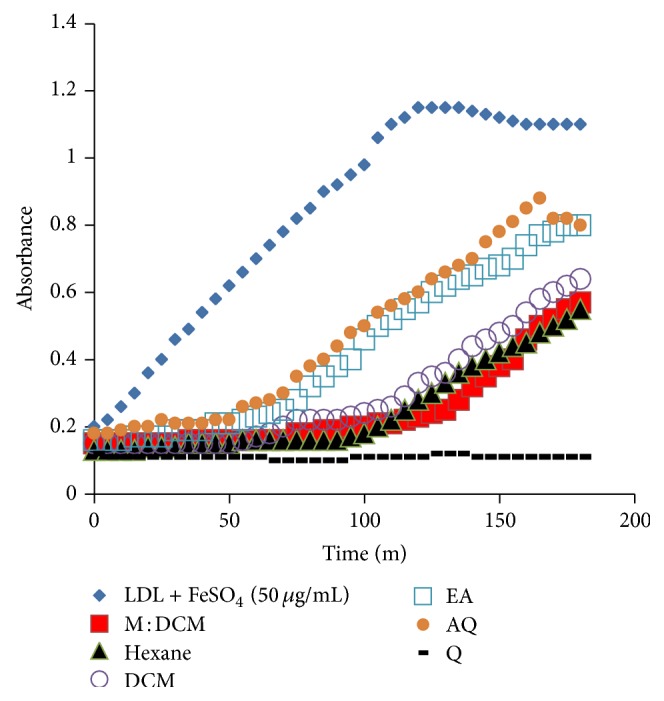
Effect of* F. velutipes* fractions upon lag time of conjugated diene (CD) formation. The gradual increase in the lag time of CD formation at 234 nm indicates the extent of ox-LDL inhibition by the respective fraction against the FeSO_4_-induced LDL oxidation. The positive control, quercetin, and all the fractions were used at 1 *μ*g/mL conc.

Fe^+2^-ions exerted peroxidative modification of the polyunsaturated fatty acids (PUFAs) present in the LDL molecule and caused molecular rearrangement, resulting in conjugated dienes (CDs) formation. As the LDL molecule's integral antioxidant, *α*-tocopherol protects LDL by withstanding the oxidative stress initially, causing a slowed oxidation, known as the lag period. Oxidation withstanding prowess of an antioxidant is directly proportional to the lengthening of the lag phase [14]. When* F. velutipes* solvent partitioned fractions were applied, the lag time was lengthened even up to 120 minutes for the M : DCM and 95 minutes for the hexane fraction, respectively (Figure 4).

#### 3.4.2. Effect of* F. velutipes* Fractions upon TBARS Formation

**Figure 5:**
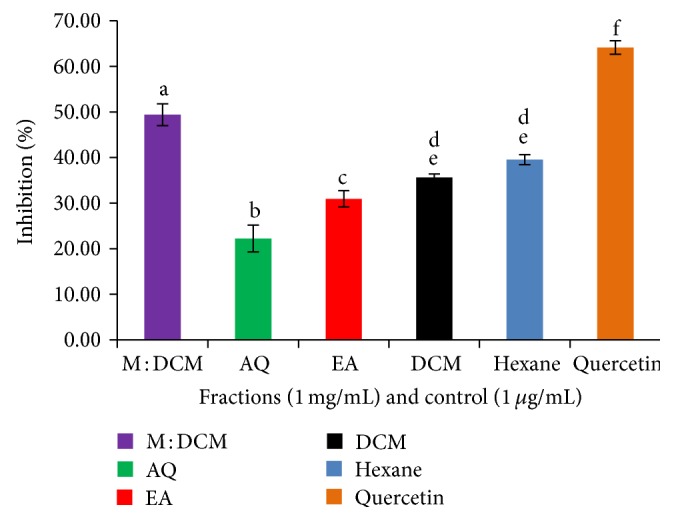
TBARS formation inhibitory effect of* F. velutipes* fractions as compared to positive control, quercetin. Data presented as mean ± SD of triplicate determinations. Mean values with different lowercase superscripts (a–f) represent statistically significant difference at 95% level (*P* ≤ 0.05) with post hoc least significance difference (LSD) test.

During LDL oxidation, the lag phase is followed by the propagation phase of rapid LDL oxidation giving rise to lipid peroxides. Lipid peroxides undergo decomposition phase when breaking of the double bonds gives rise to malondialdehydes (MDA). Nucleophilic substitution reaction between MDA and TBA used in the experimentation produced “MDA:TBA adduct,” also called “thiobarbituric acid reactive substances (TBARS)” [29]. The amount of TBARS production is inversely proportional to the antioxidant capacity of a biocomponent. All the solvent fractions of* F. velutipes *were capable of inhibiting the formation of TBARS, but the M : DCM fraction inhibited it the most (48.71%) (Figure 5).

### 3.5. Identification of Polyphenolic Compounds in the M : DCM Fraction by LC-MS/MS

**Figure 6:**
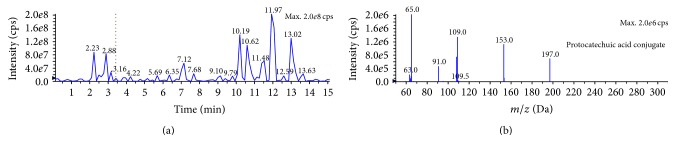
Full LC-MS/MS chromatogram (a) and the presence of protocatechuic acid in the M : DCM fraction (b) of* F. velutipes.*

**Figure 7:**
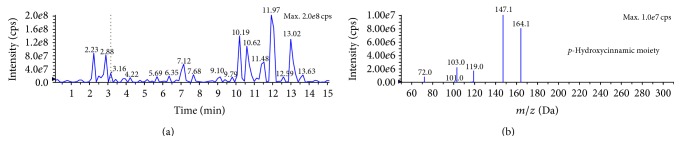
Full LC-MS/MS chromatogram (a) and the presence of* p*-hydroxycinnamic acid moiety (*p*-coumaric acid) in the M : DCM fraction (b) of* F. velutipes.*

**Figure 8:**
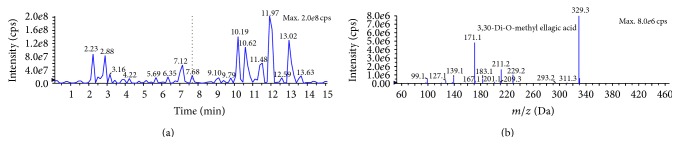
Full LC-MS/MS chromatogram (a) and the presence of ellagic acid in the M : DCM fraction (b) of* F. velutipes.*

The polyphenolic representative antioxidants present in the M : DCM fraction of* F. velutipes* were protocatechuic acid,* p*-hydroxycinnamic acid moiety (*p*-coumaric acid), and ellagic acid (Figures 6, 7, and 8 and Table 1).

#### 3.5.1. Protocatechuic Acid

Protocatechuic acid is an important polyphenolic component having therapeutic potential against oxidative stress, atherosclerosis, microbial infection, analgesic, and neurological and nephrological complications [30]. It has structural and functional similarity with other well-known antioxidants like caffeic acid, gallic acid, syringic acid, and vanillic acid [30]. Barros et al. (2009) reported the presence of protocatechuic acid in some Portuguese wild mushrooms [31]. Mattila et al. also observed the presence of protocatechuic acid in* Agaricus bisporus* and* Lentinula edodes* [32].

Protocatechuic acid is a unique antioxidant in the sense that it can prevent* in vitro* oxidative stress in both aqueous and nonaqueous media and can chelate transition metal ions and also scavenge free radicals [33]. Lende et al. (2011) reported the* in vitro* antioxidative effects of protocatechuic acid where their DPPH free radical scavenging effects and other antioxidative performances were better than the positive control, trolox [34].

Protocatechuic acid may exert antioxidative action by the following two modes.

**Figure 9:**
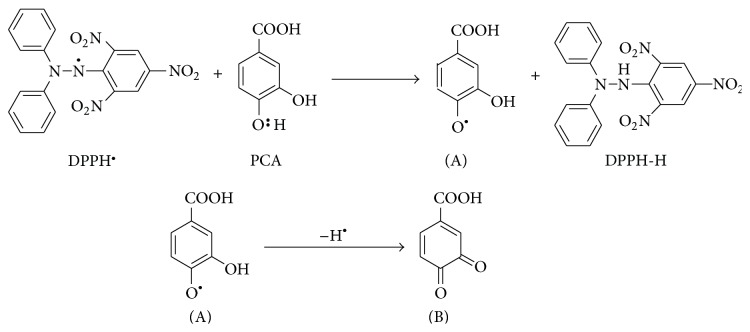


**Figure 10:**
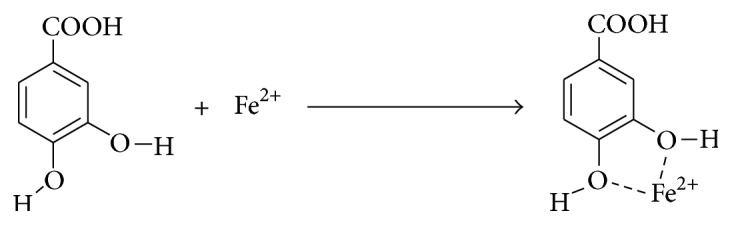


- Free radical scavenging:
  - protocatechuic acid (PCA) accepts a hydrogen atom (H^∙^) from the DPPH free radical (DPPH^∙^) forming the stable DPPH-H and the unstable anion PCA^∙^ (A);
  - the unstable anion PCA^∙^ (A) can withdraw another hydrogen atom (H^∙^) to form a stable quinone (see Figure 9).
- Metal ion chelating: the orthodihydroxyl group present in PCA aid it in chelating metal ion, in this experiment Fe^+2^ (see Figure 10).

Antiatherosclerotic effect of protocatechuic acid is also mediated via its anti-inflammatory effects. It inhibits vascular cell adhesion molecule 1 (VCAM-1) and intercellular adhesion molecule 1 (ICAM-1) expression and reduces NF-*κ*B binding activity. Thus, it prevents binding of the monocytes to the endothelial wall and further atherosclerotic processes [31]. It has been reported to attenuate platelet derived growth factor- (PDGF-) induced migration and proliferation of VSMC. The molecular mechanism involved might be the downregulation of the phosphatidylinositol 3-kinase (PI3 K)/Akt and the mitogen-activated protein kinase (MAPK) pathways [34]. It also has antihyperlipidemic effects [35].

#### 3.5.2. *p*-Hydroxycinnamic Acid Moiety (*p*-Coumaric Acid)

Barros et al. (2009) reported the presence of* p*-coumaric acid in some Portuguese wild mushrooms [31].* p*-Coumaric acid has been regarded to inhibit LDL oxidation both at* in vitro* and at* in vivo* condition [36]. The proposed mechanism involves scavenging of ^∙^OH (a representative reactive oxygen species, ROS) [36]. Its antioxidative effect upon human colon cell culture (HT-29) has also been reported [37]. Besides,* p*-coumaric acid has dually been recognized as an anti-inflammatory and platelet aggregation inhibitor in* in vivo* studies [38].

#### 3.5.3. Ellagic Acid

Ellagic acid is a flavonoid type of phenolic compound having antioxidant, anticarcinogenic, and immune-modulatory effects. Festa et al. reported the* in vitro *radical scavenging effect of ellagic acid to be stronger than that of ascorbic acid and melatonin while studying H_2_O_2_- and bleomycin-induced DNA damage [39]. Ellagic acid derivatives isolated from other sources also showed potent antioxidant effects [40]. Antioxidant studies involving colon cell lines (HCT 16) also showed similar results for ellagic acid [41]. It can enhance the production of phase II enzymes of xenobiotic metabolism and thus increase the detoxification of free radicals in hepatic tissues [42].* In vivo* studies show that ellagic acid can bind with the carcinogens and inactivate them and can prevent the damage of p53 protein [42]. Ellagic acid can aid in overcoming the host immune tolerance and has therapeutic potential for the HBV carriers [43].

During free radical-induced oxidative modification of LDL, peroxidation of polyunsaturated fatty acids generates lipid hydroperoxides. Further decomposition of hydroperoxides augments the peroxidation process until there occurs a deflection through reduction of the alkoxyl and/or peroxyl radicals to alkoxides and/or hydroperoxides, respectively. Chain-breaking phenolic antioxidants come into play in this situation and shield against the vicious cycle of oxidation and peroxidation [44]. The antioxidant capacity of phenolic antioxidants is dependent upon their structural feature, the presence of phenolic group and the potency of it to stabilize the resulting phenoxyl radical, and their redox potential: the higher the redox potential, the lower the antioxidant capacity [45]. Besides, the antioxidative effects of dietary polyphenols might be attributed to their effects upon free radical scavenging, chelating of the transition metal ions, stimulation of antioxidative enzymes, inhibition of prooxidative enzymes, and cumulative effects with other antioxidants.

**Figure 11:**
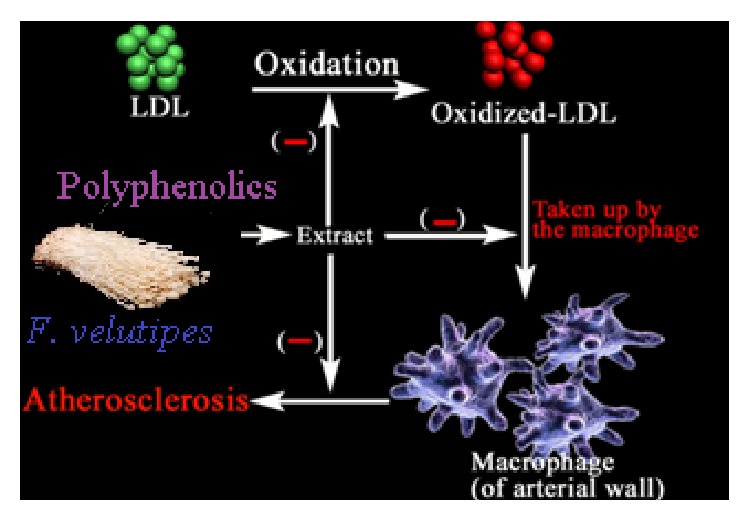
Antiatherosclerotic implications of the polyphenolic compounds of* F. velutipes*.

As described in the present study, the phenolic (ellagic acid) and polyphenolic compounds (protocatechuic acid and* p*-coumaric acid) present in the* F. velutipes* spur high in withstanding oxidative ramification of LDL that might be implicated as this mushroom's antioxidative led antiatherosclerotic accomplishment (Figure 11). Notwithstanding to surmise,* in vivo* studies incorporating these polyphenolic substances are quintessential to decipher the relevant mechanism of action.

## 4. Conclusion

As LDL oxidation is the initiating step in atherosclerotic pathogenesis, intervention at this level sounds imperative in halting the onset of atherosclerosis. Polyphenolic compounds present in the* F. velutipes* impart this mushroom's antiatherosclerotic pursuance under the aegis of antioxidative panoply. These findings, novel in case of this mushroom species, would pave a new vista for functional food-based therapeutic intervention of atherosclerosis and cardiovascular diseases and thus be of monumental importance to the humanity.

## Figures and Tables

**Table 1:** Polyphenolic compounds present in the M : DCM fraction of *F*. *velutipes*.

Number	Retention time (m)	Mode (+/−)	Compound name and structure	Molecular formula	Molecular weight (g)
1	3.4	−	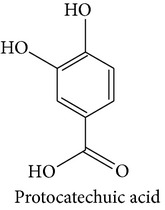	C_7_H_6_O_4_	154.12

2	5.2	−	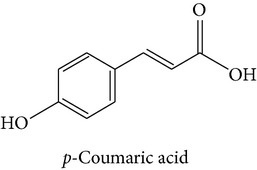	C_9_H_8_O_3_	164.16

3	7.68	−	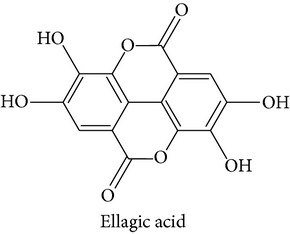	C_14_H_6_O_8_	302.197
